# TCR repertoire landscape reveals macrophage-mediated clone deletion in endotoxin tolerance

**DOI:** 10.1007/s00011-022-01685-w

**Published:** 2023-01-12

**Authors:** Juanjuan Zhao, Li Jia, YiJing Tao, Xu Zhao, Jing Yang, Yanxin Lu, Yaping Yan, Ling Mao, Lin Hu, Jia Lu, MengMeng Guo, Chao Chen, Ya Zhou, Zhenke Wen, Zhixu He, Lin Xu

**Affiliations:** 1 Special Key Laboratory of Gene Detection and Therapy of Guizhou Province, Zunyi, China; 2 Department of Immunology, Zunyi Medical University, Zunyi, 563003 GuiZhou China; 3 Department of Medical Physics, Zunyi Medical University, Zunyi, China; 4 Jiangsu Key Laboratory of Infection and Immunity, Institutes of Biology and Medical Sciences, Soochow University, Suzhou, 215123 Jiangsu China; 5 Collaborative Innovation Center of Tissue Damage Repair and Regeneration Medicine, Zunyi Medical University, Zunyi, 563000 Guizhou China

**Keywords:** Endotoxin tolerance, TCR repertoire, Clone deletion, Macrophage

## Abstract

**Background:**

Endotoxin tolerance (ET) is a protective mechanism in the process of sepsis, septic shock, and their sequelae including uncontrolled inflammation. Accumulating evidence has shown that peripheral T cells contribute to the induction of ET. However, what and how T-cell development contributes to ET inductions remain unclear.

**Methods:**

Mice were intraperitoneally injected with LPS at a concentration of 5 mg/kg to establish an LPS tolerance model and were divided into two groups: a group examined 72 h after LPS injection (72-h group) and a group examined 8 days after LPS injection (8-day group). Injection of PBS was used as a control. We performed high-throughput sequencing to analyze the characteristics and changes of CD4^+^SP TCR*β* CDR3 repertoires with respect to V direct to J rearrangement during the ET induction. Moreover, the proportion and proliferation, as well as surface molecules such as CD80 and CD86, of F4/80^+^ macrophages were analyzed using FCM. Furthermore, ACT assay was designed and administered by the tail vein into murine LPS-induced mouse model to evaluate the role of F4/80^+^ macrophages on the development of CD4^+^SP thymocytes in ET condition.

**Results:**

We found that the frequency and characteristics of the TCR*β* chain CDR3 changed obviously under condition of ET, indicating the occurrence of TCR rearrangement and thymocyte diversification. Moreover, the absolute numbers of F4/80^+^ macrophages, but not other APCs, were increased in thymic medulla at 72-h group, accompanied by the elevated function-related molecules of F4/80^+^ macrophages. Furthermore, adoptively transferred OVA_332-339_ peptide-loaded macrophages into Rag-1^−/−^ mice induced the clone deletion of OVA-specific CD4^+^SP, thereby ameliorating the pathology in lung tissue in LPS challenge.

**Conclusions:**

These data reveal that the frequency and characteristics of the TCR*β* chain CDR3 undergo dynamic programming under conditions of LPS tolerance. Furthermore, the peripheral macrophages may be a key factor which carry peripheral antigen to thymic medulla and affect the negative selection of T-cell population, thereby contributing to the formation of ET. These results suggest that the clone selection in thymus in ET may confer protection against microbial sepsis.

**Supplementary Information:**

The online version contains supplementary material available at 10.1007/s00011-022-01685-w.

## Introduction

Sepsis, septic shock, and their sequelae including uncontrolled inflammation are the leading causes of death in intensive care units, with limited therapeutic options. Pathophysiological adaptations to regulate over-exuberant inflammation serve as an important mechanism for host protection against endotoxin shock. One of these protective mechanisms is endotoxin tolerance (ET) [1, 2]. Long-term exposure to lipopolysaccharide (LPS) or injection of sub-lethal doses of LPS in animals can induce tolerance of endotoxin that reprograms the inflammatory response, resulting in cells or organisms entering into a transient unresponsive state where they are unable to respond to further challenges with endotoxin. In recent years, the studies about ET mainly focus on the innate cells, such as macrophages, and innate molecule TLR4-related signaling pathways [3]. Meanwhile, some factors, such as TLR2, Gi protein, and PKC, are also responsible for the induction of ET. Interesting, a growing number of studies reported that adaptive immune cells, such as T cells, were also contributed to ET induction. T-cell depletion mediates the decreased sensitivity to LPS in TGF-β^−/−^ mice or SCID mice, which prolongs the survival of several autoimmune disease mice [4, 5]. Moreover, pathologic CD4^+^T cells contribute to exaggerated immune activation toward LPS challenge that impairs the induction of ET [6]. These research works supported that T cells play a critical role for the formation of ET.

It is deserved to note that the development of T cells is closely related to the formation of central tolerance and contributes to the process of various diseases. For example, the proportion of thymocytes is abnormal under conditions of Leishmania infection combined with malnutrition [7]. In addition, the migration of T cells to peripheral immune organs increases during acute infection with Trypanosoma cruzi, which affects thymocyte development [8]. Similarly, a study has reported that a reduction in thymic emigrants contributes to the development of coronary heart disease, which may be related to the destruction of immune tolerance caused by T-cell confusion and thymus degeneration [9]. Interestingly, our recent studies showed that the total numbers and function of thymocytes were changed under endotoxin tolerance induction [10]. These findings raise an interesting question: what and how T-cell development contributes to ET induction and urged a re-evaluation as the new mechanism of ET.

In the present study, we aimed to evaluate the role of thymocyte development in ET establishment from the perspective of central tolerance based on the high-throughput analysis of T-cell receptor (TCR) repertoire. We found that there was the change of CD4^+^SP TCR*β* CDR3 repertoires during the induction of ET. Of note, peripheral macrophages may be as a key factor which carry peripheral antigen to thymic medulla and interaction with thymocytes contributes to negative selection of T-cell population, subsequently participating in the formation of ET. These key points may be helpful for the development of therapeutic approaches of uncontrolled inflammation in Sepsis.

## Materials and methods

### Mice and model

C57BL/6 wild-type (WT) mice, Rag1^−/−^ mice, and OT-II mice (female, 8–10 weeks of age) [11] were housed under specific pathogen-free (SPF) conditions at Zunyi Medical University, according to the guidelines for the Care and Use of Laboratory Animals (Ministry of Health, China, 1998). The experimental procedures were approved by the Zunyi Medical University Laboratory Animal Care and Use Committee (permit number SYXK2013068).

LPS tolerance model: female WT mice at 8 to 10 weeks of age were intraperitoneally injected with LPS at a concentration of 5 mg/kg to establish an LPS tolerance model, and two groups were established: a group examined 72 h after LPS injection (72-h group) and a group examined 8 days after LPS injection (8-day group). PBS was injected for the control group (control group). Thymus tissue was obtained from all mice at the indicated times.

ACT model: Bone marrow-derived monocytes (BMDMs) purified from WT mice (female 8–10 weeks) were cultivated 7 days with GM-CSF (20 ng/ml). Then, these cells were labeled with CFSE (5 μM). 2 × 10^6^ cells were adoptively transferred into syngenic WT mice through tail vein. 24 h later, mice were treated with 5 mg/kg LPS. Next, the distribution of CFSE^+^ macrophages was observed by image assay at the indicated point times.

Attack experiment of high-dose LPS model: Bone marrow-derived from OT-II mice was adoptively transferred into syngenic Rag1^−/−^ mice through tail vein. 24 h later, mice were injected with OVA_332-339_-loaded macrophages (2 × 10^6^ cells) *i.v.* and then treated with 0.5 mg/kg LPS *i.p.*. The distribution of OVA-specific CD4^+^ T cells was observed and analyzed by FCM using Tetramer technique. 72 h later, mice were retreated with 2 mg/kg LPS. Thymus tissue was obtained from all mice at the indicated times.

### High-throughput sequencing

Next-generation sequencing of TCR was carried out as previously described [12]. We obtained thymic CD4^+^SP cells from mice in different groups using MACS. Briefly, DNA was extracted from these thymic CD4^+^ SP cells using DNeasy Blood &Tissue kit (Qiagen), quantified using a Qubit Fluorometer (Thermo) and amplified by multiplex-PCR of rearranged variable, diverse, joining (VDJ) segments of the TCR genes, which encode the hypervariable CDR3 domain. The products were size selected using Pronex beads (Promega) and subsequently sequenced on a MiSeq (Illumina). The length and polymorphism of CDR3 were analyzed with GeneMapper 4.1 software (Thermo).

### Histopathology

Indicated tissues were fixed in 4% paraformaldehyde, embedded in paraffin, and cut into 3.5-μm-thick sections. Sections were stained with H&E, and images were taken with an Olympus IX71 microscope. Two investigators blinded to group assignments analyzed the samples and determined the injury levels.

### Immunofluorescence (IF)

Sections were hydrated and rinsed with PBS three times (5 min each) and then blocked with 10% normal goat serum at room temperature for 10 min and incubated with rabbit anti-mouse antibodies at appropriate dilution in TBS overnight at 4 °C. The primary antibodies used were as follows: FITC-UEA-1 (1:50; Vector Laboratories; no. FL-1061–2) and F4/80 (1:250; Abcam; no. ab204467). PBS instead of primary antibody served as a control. Then, the slices were rinsed with cold PBS three times (5 min each). Finally, the sections were mounted with Slow Fade Gold Antifade Reagent with DAPI and examined by fluorescence microscopy.

### Flow cytometry

Cytokines, transcriptional factors, and surface markers of various immune cells were evaluated by flow cytometry (FCM) with Beckman Gallios (Beckman Coulter, Inc.). FCM was performed on Beckman Gallios (Beckman Coulter, Inc.) with CellQuest Pro software using directly conjugated mAbs against the following markers: F4/80-Percp-Cy5.5 (45–4801-82), MHC-II-APC (no. 17–5320-82), CD86-Percp-Cy7 (no. 25–0862-82), CD80-PE (no. 12–0801-82), CD11c-PE (no. 12–0114-82), CD19-Percp-Cy7 (no.25–0193-82), NK1.1-APC (no. 17–5941-81), CD4-Percp-Cy5.5 (no. 12–0041-82), CD62L-PE (no. 12–0621-81), CD69-APC (no. 17–0691-82), IFN-γ-Percp-Cy5.5 (no. 45–7311-82), IL-4-APC (no. 17–7041-82), and Ki67-PE (no. 12–5698-82), with corresponding isotype-matched controls (eBioscience). For intracellular staining, cells were first surface stained for activated markers and then fixed, permeabilized with IC Fixation & Permeabilization buffer (eBioscience, 88–8823-88), and stained with antibodies against IFN-γ, IL-4, Ki67 (eBioscience), respectively, at 4 ℃ for 30 min in dark. After washing twice, stained cells were analyzed with a Beckman coulter flow cytometer.

The live status of cells was first analyzed by trypan blue staining. Under the condition of the percentage of live cells was above 90%, cells then were analyzed in following experiments. The absolute numbers of thymocytes were counted by cell counting chamber. The percentage of various subpopulations including F4/80^+^ macrophages, CD19^+^ B cells, NK1.1^+^ cells, and other cells in thymocytes were analyzed using flow cytometry (Beckman Coulter, USA), and then, the absolute numbers of these cell populations were calculated. Finally, the percentages of CD86, CD80, and MHC class II were analyzed based on the gating on F4/80^+^ macrophages (Sfig.3).

### Statistical analyses

The data were analyzed with GraphPad Prism 7.0 and are presented as the mean ± SD. Student’s t test was used when two conditions were compared, and analysis of variance with Bonferroni or Newman–Keuls correction was used for multiple comparisons. Probability values of < 0.05 were considered significant; two-sided tests were performed.

## Results

### The frequency and characteristics of CD4^+^SP TCRβ CDR3 repertoires undergo dynamic programming during the ET induction

**Fig. 1:**
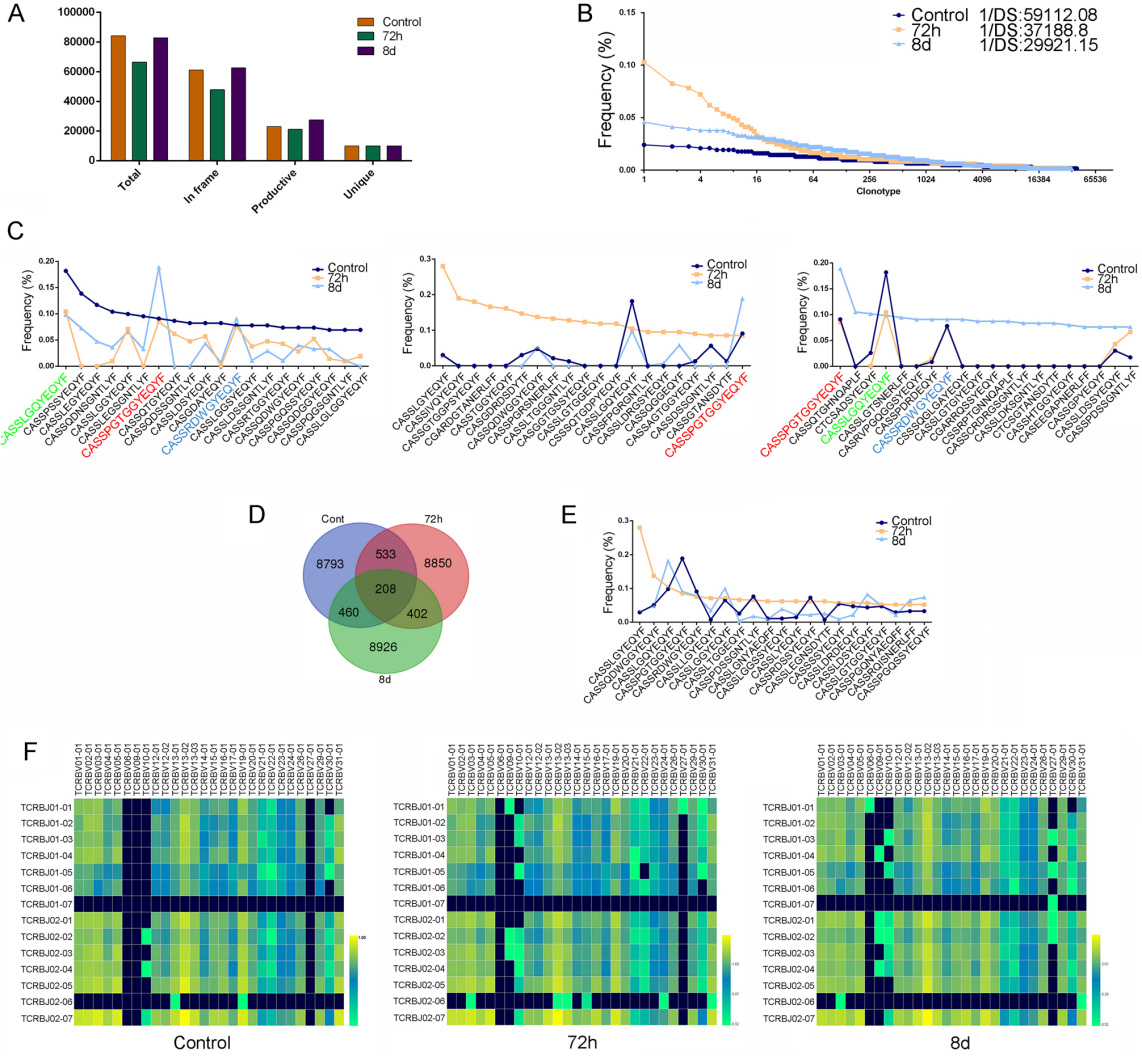
The change of thymic CD4^+^SP TCRβ chain in endotoxin tolerance. The C57BL/6 WT mice (female *n* = 9) were treated with 5 mg/kg LPS *i.p.*. At indicated time points (0 day, 72 h, and 8 days), the CDR3 repertoires of thymic CD4^+^SP cells purified by MACS were analyzed using high-throughput sequencing, respectively. **A** The total sequences, unique sequences, and in frame sequences, and clonotype distribution plots **B**, AA usage of TCR beta chain V–J rearrangement and CDR3 repertoire with TRBV and TRBJ pairing were analyzed **C–F**

TCR repertoires can characterize the features of the host T-cell immune status [13]. In particular, dynamic analysis of the TCR repertoires of T cells is valuable for estimating the immune reconstitution in the host with different situations, which play an important role in the assessment of multiple factors such as tumor, inflammatory, and vaccine-mediated immune response and the development of future therapeutics [14, 15]. Our previous results showed that the total numbers and function of thymocytes, especially CD4^+^SP, were changed significantly under ET conditions [10], Therefore, in the present study, we performed high-throughput sequencing to analyze the characteristics and changes of CD4^+^SP TCR*β* CDR3 repertoires with respect to V direct to J rearrangement during the ET induction. We found that the total and in frame sequence amounts of V–J rearrangement in the CDR3 repertoire in 72 h of post-LPS *i.p.* group were significantly reduced, but the proportion of clonotype exhibited increased, compared with those in control group and 8 days of post-LPS *i.p.* group (Fig. 1A and B). Furthermore, a Gaussian CDR3 length distribution pattern was also observed during ET induction. The amino acids’ (AA) length of TCR beta chain V–J rearrangements in the TCR*β* CDR3 repertoire was between 6 and 23 aa, and the highest peak was 12 aa. In AA usage of TCR*β* chain V–J rearrangements in the CDR3 repertoire, the usage of I and V amino acids decreased in 72 h of post-LPS *i.p.* group; In contrast, R, D, and K were the dominant amino acids in 8 days of post-LPS *i.p.* group. In addition, W, Y, and V were also reduced in V–J rearrangements in the CDR3 repertoire of 8 days after LPS treatment (Sfig.1A-D).

TRBV, TRBJ usage, and TRBV–TRBJ gene pairing of the TCR*β* chain V–J rearrangements in the CDR3 repertoire were also analyzed (Fig. 1C–F). At 72 h of post-LPS *i.p.*, the preferential use of TRBV in the CD4^+^SP TCR*β* chain V–J rearrangement in the CDR3 repertoire involved TRBV01-04 and TRBJ02-02 (Fig. 1F). The top 3 of TRBV-TRBJ preferential gene pairings were TRBV13-02/TRBJ02-06, TRBV13-03/TRBJ01-02, and TRBV10-01/TRBJ01-02, compare with control group and 8 days of post-LPS *i.p.* group (Fig. 1F). These data suggested that the frequency and characteristics of the TCR*β* chain CDR3 undergo dynamic programming under conditions of LPS tolerance, indicating that there were clonal selection or deletion of T-cell repertoire with a specific TCR*β* chain V–J rearrangement in the CDR3 repertoire during ET induction.

### The peripheral macrophages migrated into thymus in ET condition

**Fig. 2:**
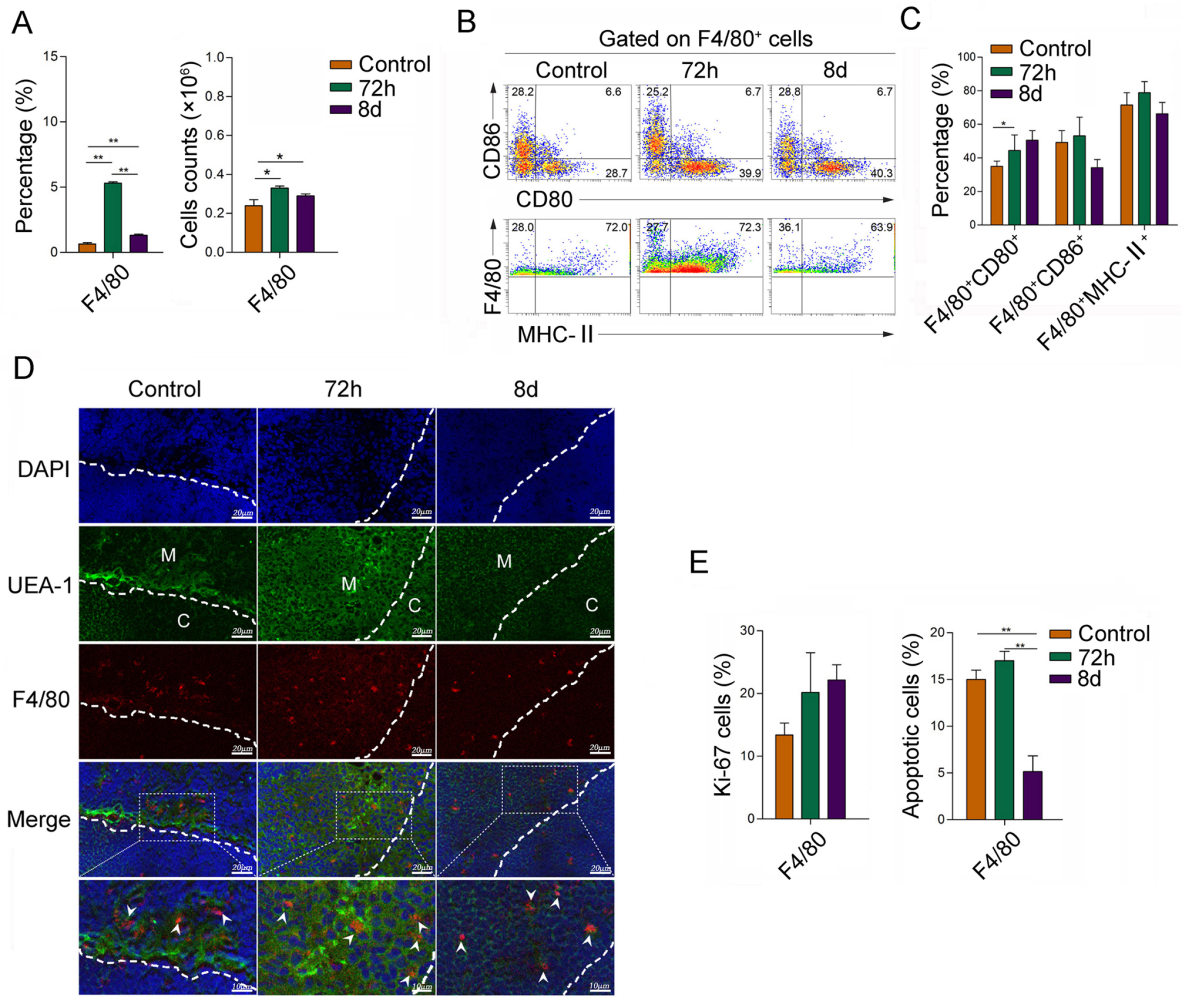
The change on macrophages in thymus in endotoxin tolerance. The C57BL/6 WT mice (female *n* = 9) were treated with 5 mg/kg LPS *i.p.*. At indicated time points (0 day, 72 h and 8 days), the percentage and cell numbers of F4/80^+^ macrophages **A** were analyzed and calculated. The gating strategy was shown in supplementary Fig. 3. **B–C** The activation-associated molecules CD80, CD86, and MHC-II, as well as the proliferation-related molecule Ki-67 and apoptosis **E** of F4/80^+^ macrophages in thymus were detected by FCM and calculated, respectively. **D** The localization of F4/80^+^ macrophages was detected by Immunofluorescence confocal. UEA-1 (green) immunostaining of medulla, DAPI (blue), and F4/80^+^ macrophages (red). White line indicates the region between thymic medulla and cortex. The values are the means ± SD (*n* = 9). *P < 0.05, **P < 0.01 (color figure online)

The professional antigen-presenting cells (APC) are a class of adjuvant cells that processing and presenting antigen information to affect T-cell development in thymus, including dendritic cells (DCs), monocytes/macrophages, and B lymphocytes [16, 17]. Recent studies have shown that the professional APCs directional migration is not only important for immune response and regulation, but also cause the developing antigen-specific T cell to clonal deletion to take part in the process of negative selection for maintaining central tolerance in the thymus medulla region [18]. Herein, we further showed that, compared with that in control group, the absolute numbers of F4/80^+^ macrophages were increased in 72 h of post-LPS *i.p.* group*.* (Fig. 2A), not other APCs, such as CD11c^+^ DCs, CD19^+^ B cells, and NK1.1^+^ cells (Sfig.2A-E). FCM analysis showed that, compared with the control group, the expression level of CD80 on F4/80^+^ macrophages increased, even the levels of CD86 and MHC class II increased mildly at 72 h of post-LPS *i.p.* (Fig. 2B and C). Immunofluorescence data further confirmed that F4/80^+^ macrophages were increased in thymus at 72 h of post-LPS *i.p*., which mostly were located in thymic medulla (Fig. 2D). Unexpectedly, compared with control group, the apoptosis of F4/80^+^ macrophages were obviously increased at 72 h of post-LPS *i.p.*, whereas the proportion was decreased at 8 days of post-LPS *i.p.* (Fig. 2E). Meanwhile, the proliferation of F4/80^+^ macrophages did not change significantly (Fig. 2E). In additions, the absolute numbers of F4/80^+^ macrophages in peripheral immune organ spleen were prominently decreased (data not shown). These data indicate that the migration of peripheral macrophages into thymus, but not resident macrophages, might be main responsible for the macrophage population enrichment in thymus in ET condition.

**Fig. 3:**
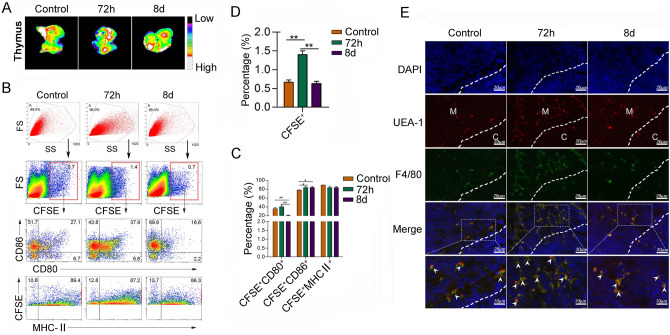
The peripheral macrophages migrate into thymus in endotoxin tolerance. Bone marrow-derived monocytes (BMDMs) purified from WT mice were cultivated 7 days with GM-CSF (20 ng/ml). Then, cells were labeled with CFSE. 2 × 10^6^ cells were adoptively transferred into syngenic WT mice through tail vein. 24 h later, mice were treated with 5 mg/kg LPS. Next, the distribution of CFSE^+^ macrophages was observed by image assay **A**. The proportion of CFSE^+^ macrophages, and the activation-related molecule CD80, CD86, and MHC-II of CFSE^+^ macrophages in thymus were analyzed by FCM and calculated **B-D**. **E** The localization of CFSE^+^ macrophages was detected by Immunofluorescence confocal. One representative data of three independent experiments were shown. **P < 0.01

To confirm the migration of peripheral macrophages into thymus in ET condition, we adoptively transferred CFSE labeling F4/80^+^ macrophages into normal mice through tail vein. Next, 24 h later, these mice were challenged with 5 mg/kg LPS *i.p.*. In vivo fluorescence imaging and FCM analysis showed that the infiltrating CFSE^+^ macrophages in thymus tissues markedly increased at 72 h and decreased at 8 days of post-LPS *i.p.* compared with the 0 day of post-LPS *i.p.* (Fig. 3A–D). Moreover, these cells dominantly located in thymic medulla (Fig. 3E). Furthermore, the expression levels of CD86 and CD80 on CFSE^+^ macrophages increased at 72 h of post-LPS *i.p.*, even the level of MHC class II did not change at 72 h and 8 days of post-LPS *i.p.* (Fig. 3B–C). These data suggested that macrophages, but not other APCs, might migrate into thymus and affect the T-cell development in ET condition.

### The peripheral macrophages mediated negative selection of T-cell population

**Fig. 4:**
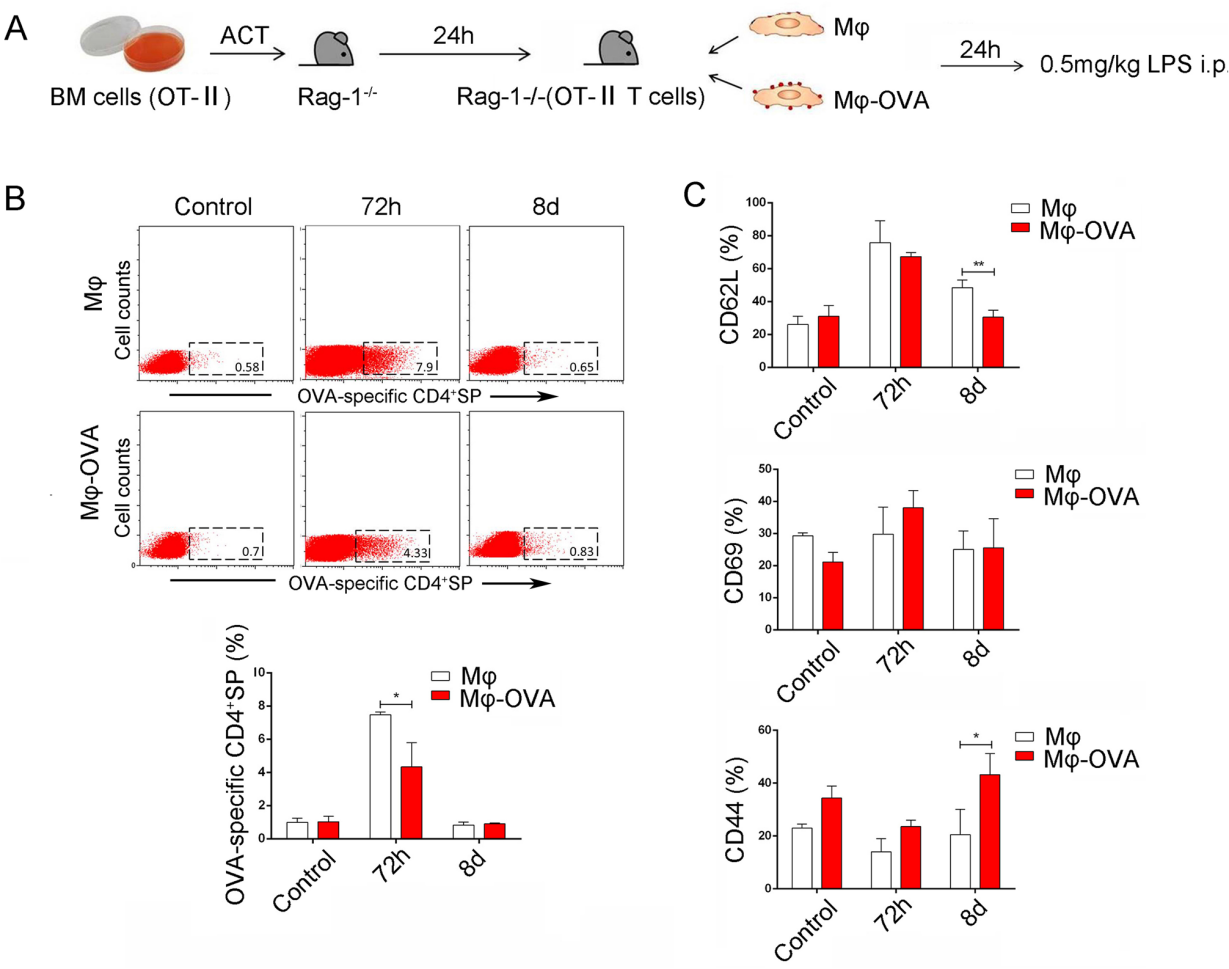
The peripheral macrophages mediate negative selection of T-cell population. **A** Bone marrow cells derived from OT-II mice were adoptively transferred into syngenic Rag1^−/−^ mice through tail vein. 24 h later, mice were injected with OVA_332-339_-loaded macrophages (2 × 10^6^ cells) *i.v.* and then treated with 0.5 mg/kg LPS *i.p.*. The distribution of OVA-specific CD4^+^ T cells and its activation-related molecule CD62L, CD44 and CD69, was observed and analyzed by FCM using Tetramer technique **B–C**. The values are the means ± SD (*n* = 9). *P < 0.05, **P < 0.01

TCR gene, especially CDR3 region, happens rearrangement in T-lymphocytes produce some autoreactive TCR which could interact high affinity with APCs presenting autoantigen–MHC complex to start the apoptosis program results in clone deletion. Our above data showed that there were change of TCR*β* CDR3 repertoire of CD4^+^SP cells in the condition of ET. Next, to further explore whether peripheral macrophages migrated into thymus and affected T-cell development, we further adoptively transferred OVA_332-339_ peptide-loaded macrophages into Rag-1^−/−^ mice (the mice were pre-transferred with bone marrow cells from OT-II mice). After 24 h, these mice were injected with 0.5 mg/kg LPS *i.p.* (Fig. 4A). Data showed that the proportion of OVA-specific CD4^+^SP in thymus were obviously increased at 72 h and decreased at 8 days of post-LPS treatment in control unloaded macrophage transferred group (Fig. 4B), which were similar to our previous results [10]. Importantly, the proportion of thymic OVA-specific CD4^+^SP were obviously decreased at 72 h of post-LPS treatment in the OVA_332-339_ peptide-loaded macrophage transferred group (Fig. 4B, C). Meanwhile, the expression level of CD62L on these thymic OVA-specific CD4^+^SP decreased and the expression level of CD44 increased, even the expression of CD69 did not change (Fig. 4B, C). These data indicated that there were clonal deletion on OVA-specific CD4^+^SP in thymus during ET induction.

### Macrophage-mediated clone deletion ameliorates the pathology of LPS-induced sepsis

**Fig. 5:**
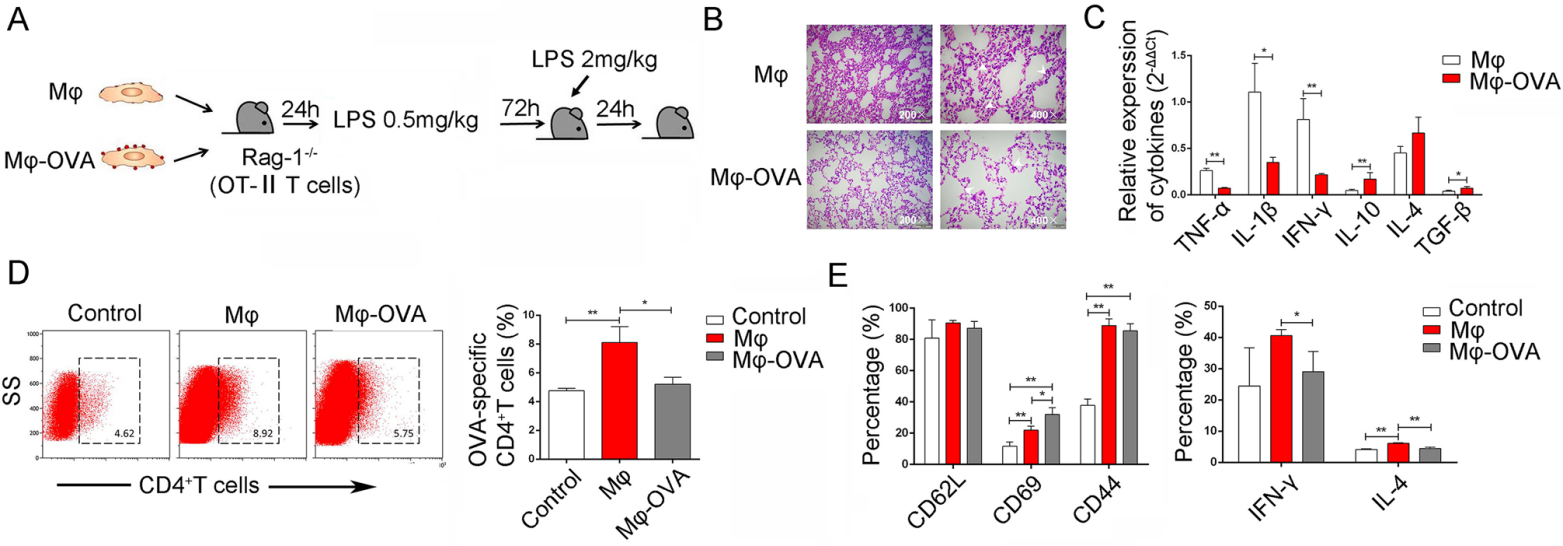
Macrophage-mediated clone deletion ameliorates the pathology of LPS-induced sepsis. Bone marrow cells derived from OT-II mice were adoptively transferred into syngenic Rag1^−/−^ mice through tail vein. 24 h later, mice were injected with OVA_332-339_-loaded macrophages (2 × 10^6^ cells) *i.v.* and then treated with 0.5 mg/kg LPS *i.p.*. 72 h later, mice were retreated with 2 mg/kg LPS *i.p.*. The pathology of lung tissue was detected **A** and the expression levels of inflammatory cytokines TNF-α, IL-1β, IFN-γ, IL-10, IL-4, and TGF-β were analyzed by real-time PCR and calculated **C**. The proportion and functionally associated molecules CD62L, CD69, and IFN-γ in OVA-specific CD4^+^ T cells in thymus were analyzed by FCM and calculated **D–E**. The values are the means ± SD (n = 9). *P < 0.05, **P < 0.01

To further explore the possible effect of clonal deletion of OVA-specific CD4^+^SP on ET induction, we observed the pathological damage of lung in recipient mice after a lethal dose of LPS administration. Expectedly, data showed that there was ameliorated pathology in lung tissue in the OVA_332-339_ peptide-loaded macrophage transferred group (Fig. 5A and B). Consistently, the expression levels of pro-inflammatory factors such as TNF-α, IL-1β, and IFN-γ in lung tissue also decreased significantly (Fig. 5C); conversely, the expression levels of anti-inflammatory factors, such as IL-10 and TGF-β, increased obviously, even though IL-4 only tended to be upregulated (Fig. 5C). Further analysis showed that the proportion of peripheral OVA-specific CD4^+^T cells also decreased (Fig. 5D). Finally, even though the expression level of CD62L did not change, the expression level of CD69 increased significantly in OVA-specific CD4^+^T cells, displaying a hyperactivation status. However, the expression of IFN-γ in these CD4^+^T cells was markedly reduced (Fig. 5E). Collectively, our data demonstrated that peripheral macrophages carry peripheral antigen to thymic medulla, and then, interaction with thymocytes contributes to negative selection of T-cell population, subsequently participating in the formation of ET and affecting lung injury development.

## Discussion

A large number of recent studies have shown that the secretion of inflammatory cytokine and mortality are decreased in the state of ET [19, 20]. Moreover, the proportion and function of T cells play a decisive role for the formation of ET [10, 21, 22]. However, what and how T-cell development contributes to ET remain to be fully elucidated. TCR repertoires can characterize the features of the host T-cell immune status. For example, Simpson [23] showed that the TCR repertoire of cytomegalovirus (CMV)-specific CD8^+^ T cells to establish if TCR tissue-specificity was shared among viruses that chronically replicate. Rowntree’s TCR sequencing data showing reduced clonal expansion in unvaccinated children who seroconverted had comparable Spike-specific but lower ORF1a- and N-specific memory T-cell responses compared with adults [24]. Similarly, in the present study, our data showed that the frequency and characteristics of the TCR*β* chain CDR3 undergo dynamic programming under conditions of LPS tolerance, indicating that there were clonal selection or deletion of T-cell repertoire with a specific TCR*β* chain V–J rearrangement in the CDR3 repertoire during ET induction. Consistently, our recent study showed that the total numbers and function of thymocytes were changed under ET induction. Therefore, our current findings further support the fact that there is the change on T-cell development in ET condition.

A large number of recent studies have shown that the professional APC, including DCs, macrophages and B lymphocytes, directional migration is not only important for immune response and regulation, but also could migrate to the thymus to participate in the development of thymocytes. Such as, Bonasio [25] found that circulating DCs through kinds of adhesion molecules migrate to the thymus medulla, causing the developing antigen-specific T cell to clonal deletion to take part in the process of negative selection for maintaining central tolerance. Herein, we found that the proportion and absolute numbers of F4/80^+^ macrophages, not other CD19^+^ B cells and DCs, were increased at 72 h of post-LPS *i.p*. Moreover, the expression level of CD80 on these F4/80^+^ macrophages were obviously increased. Importantly, the increased F4/80^+^ macrophages in thymus were mostly located in thymic medulla, indicating that macrophages might be the main responsible for the change on T-cell development in ET. In addition, we noticed that the proportion and the absolute numbers of F4/80^+^ macrophages in spleen were prominently decreased. Thus, combining these data indicated that the migration of peripheral macrophages into thymus, but not resident macrophages, might be main responsible for the macrophage population enrichment in thymus in ET condition.

Post-positive selection SP migrate into the thymic medulla to undergo negative selection to acquire autoimmune tolerance, suggesting that thymus macrophages are strongly linked to the establishment of the central tolerance in LPS-induced acute inflammation. In the present study, we further reconstructed the thymus development system of Rag-1^−/−^ mice and found that OVA-loaded macrophages could not only migrate into the thymus but also affect the development of OVA-specific CD4^+^T cells in ET condition. Importantly, we revealed that there was ameliorated pathology of lung tissue after a lethal dose of LPS administration in the OVA-loaded macrophage transferred group. In line with these findings, we further found that the proportion and IFN-γ secretion of peripheral OVA-specific CD4^+^ T cells also decreased. Collectively, our data indicated that peripheral macrophages carry peripheral antigen to thymic medulla, and then, interaction with thymocytes contributes to negative selection of T-cell population, subsequently participating in the formation of ET and affecting lung injury development.

## Conclusion

In all, we found that there was the change of CD4^+^SP TCR*β* CDR3 repertoires during the induction of ET. Of note, peripheral macrophages may be as a key factor which carry peripheral antigen to thymic medulla and interaction with thymocytes contributes to negative selection of T-cell population, subsequently participating in the formation of ET. These findings might provide a new basement for the explorations on the establishment of ET and would have a profound understanding to the protective mechanism for some acute disorders and benefit the outcome of related clinical diseases.

## Supplementary Information

Below is the link to the electronic supplementary material.

Supplementary file1 (PDF 652 KB)

## Data Availability

The datasets analyzed during this study are available from the corresponding author on reasonable request.

## Abbreviations

APC: Antigen-presenting cells
ACT: Adoptive cell transfer
BMDMs: Bone marrow-derived monocytes
CDR3: Complementarity-determining region 3
ET: Endotoxin tolerance
LPS: Lipopolysaccharide
TCR: T-cell receptor
TRBV: T-cell receptor beta variables
