# Supplementary material for: TCR repertoire landscape reveals macrophage-mediated clone deletion in endotoxin tolerance
Source: Inflamm Res. 2023 Jan 12;72(3):531–40. doi: 10.1007/s00011-022-01685-w (PMC10023648; doi:10.1007/s00011-022-01685-w)
Supplement: Supplementary file 1 — Supplementary file1 (PDF 652 KB) [file 11_2022_1685_MOESM1_ESM.pdf]

TCR repertoire landscape reveals macrophage-mediated clone deletion in endotoxin tolerance

Juanjuan Zhao[1,2+*], Li Jia[1,2+], YiJing Tao[1,2+], Xu Zhao[1,2], Jing Yang[1,2], Yanxin Lu[1,2], Yaping Yan[1,2], Ling Mao[1,2], Lin Hu[1,2], Jia Lu[1,2], MengMeng Guo[1,2], Chao Chen[1,2], Ya Zhou[3], Zhenke Wen[4,*], Zhixu He[5,*], Lin Xu[1,2,*]

[1] Special Key Laboratory of gene detection & therapy of Guizhou Province, Zunyi, China

[2] Department of Immunology, Zunyi Medical University, Zunyi, China

[3] Department of Medical physics, Zunyi Medical University, Zunyi, China

[4] Jiangsu Key Laboratory of Infection and Immunity, Institutes of Biology and Medical Sciences, Soochow University, Suzhou, China

[5] Department of Paediatrics, Affiliated hospital of Zunyi Medical University, Zunyi, China

[+] These authors contributed equally to this work.

*** Correspondence to:**

Dr. Lin Xu, Department of immunology, Zunyi Medical University, Zunyi, GuiZhou 563003, China, Email: xulinzhouya@163.com.

Dr. Zhixu He, Department of Paediatrics, Affiliated hospital of Zunyi Medical University, Zunyi, Guizhou 563000, China, Email: hzx@gmc.edu.cn

Dr. Zhenke Wen, Jiangsu Key Laboratory of Infection and Immunity, Institutes of Biology and Medical Sciences, Soochow University, Suzhou, Jiangsu 215123, China. Email: zkwen@suda.edu.cn

Dr. Juanjuan Zhao, Department of immunology, Zunyi Medical University, Zunyi, GuiZhou 563003, China, Email: jj.z.2008@163.com.

Number of Figures: 5

Number of supplementary Figures: 3

Supplementary Figure 1-3

A

Length distribution frequency of TCR beta chain CDR3 repertoire from three groups

	Control	72h	8d
4	0	0	0.008700948
5	0.01623772	0	0.008700948
6	0.01623772	0.042197654	0.026102845
7	0.146139482	0.067516246	0.191420865
8	0.925550053	0.77643683	0.913599582
9	3.929528294	4.523588488	3.628295484
10	12.85215556	11.07266436	9.301313843
11	22.35122189	20.53337834	18.01096319
12	31.78533734	31.90986581	29.9660663
13	19.71259235	21.13258503	24.20603846
14	6.592514411	7.730610178	10.07569825
15	1.250304457	1.780740991	2.784303489
16	0.324754404	0.3291417	0.635169233
17	0.064950881	0.092834838	0.191420865
18	0.03247544	0.008439531	0.043504742
20	0	0	0.008700948

C

AA usage distribution of TCR beta chain CDR3 repertoire from three group

AA	Cont-1	72h-1	8d-2
A	11.64670535	11.88070811	11.5751514
R	4.219441267	4.153684916	5.039964155
N	4.140193227	4.050306215	4.6923555
D	4.679769009	4.935085305	5.198230882
C	0.520280607	0.541846985	0.61283189
Q	7.78284659	7.895993897	7.320739445
E	6.102099039	5.933224489	6.029311865
G	11.70527999	11.8792822	11.24850044
H	0.387281723	0.371450368	0.48202697
I	0.753200932	0.670179166	0.816627401
L	6.376366167	6.36598912	6.282972235
K	0.354204281	0.349348714	0.475522858
M	0.121283956	0.137600616	0.117074017
F	2.200339044	2.17594342	2.350152485
P	2.293369351	2.434033694	2.821339269
S	18.75284259	18.33724271	18.45072051
T	6.06971071	6.390229643	6.055328313
W	1.205259313	1.336793549	0.904071574
Y	9.484267541	9.25275023	8.694552445
V	1.205259313	0.908306657	0.832526342

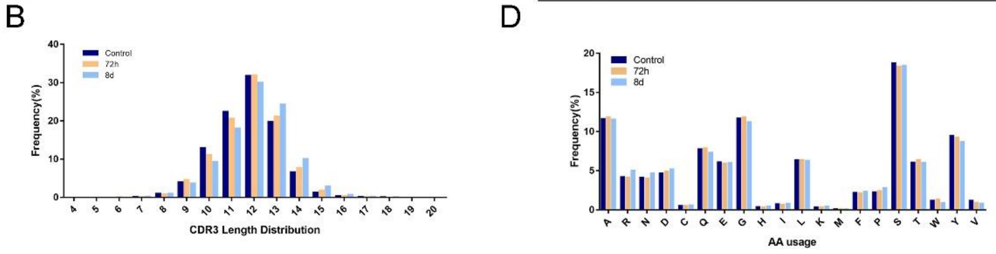

Supplementary Figure 1. The change of AA length distribution and AA usage of V-J rearrangement in the thymic CD4⁺SP TCRβ CDR3 repertoires under the condition of endotoxin tolerance.

The C57BL/6 WT mice (female, n=9) were treated with 5 mg/kg LPS *i.p.*. At indicated time point (0 day, 72 hours and 8 days), thymic CD4$^+$SP cells purified by MACS were analyzed using high-throughput sequencing respectively. **(A-B)** AA length distribution and **(C-D)** AA usage of V-J rearrangement in the TCRβ CDR3 repertoire were analyzed.

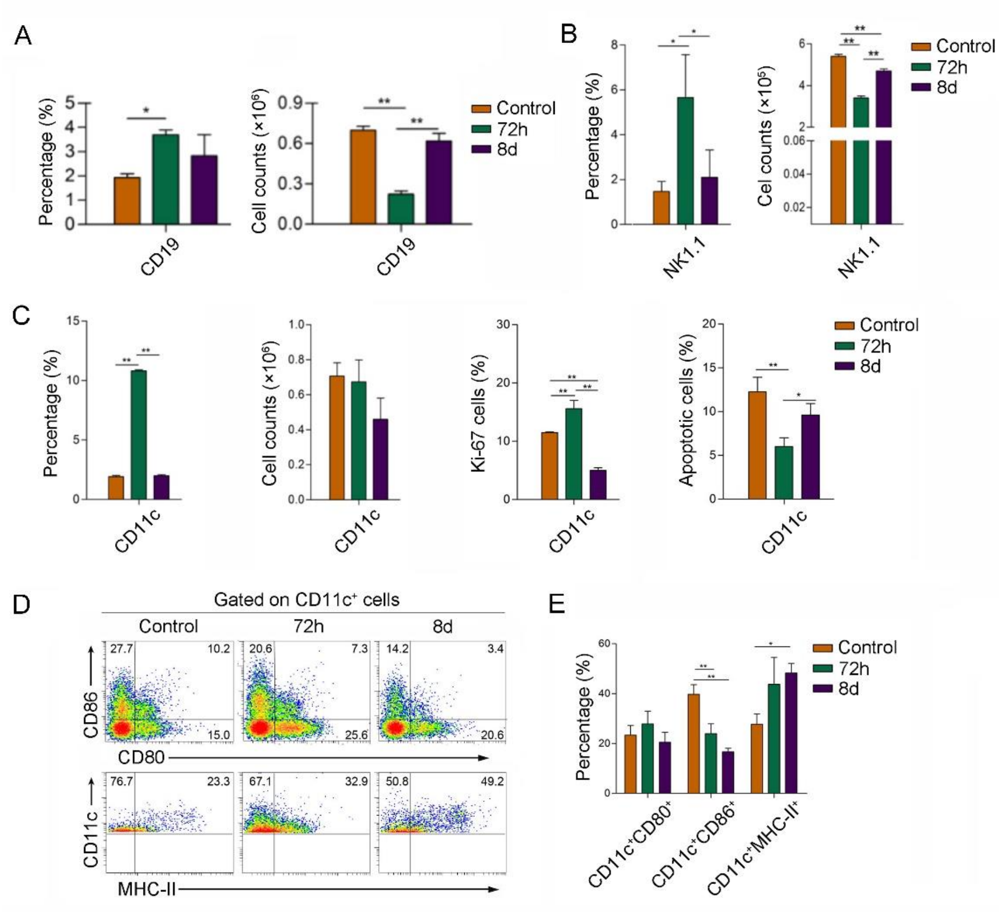

Supplementary Figure 2. The change of antigen-presenting cells in thymus under the condition of endotoxin tolerance.

The C57BL/6 WT mice (female, n=9) were treated with 5 mg/kg LPS *i.p.*. At indicated time points (0 day, 72 hours and 8 days), (A-C) The percentage and cell numbers of antigen-presenting cells (APCs) such as CD11c$^+$ cells, CD19$^+$ B cells and NK1.1$^+$ cells in thymus were detected by FCM and calculated. The expression of proliferation-related molecule Ki-67, activation-related molecule CD86, CD80 and MHC-II and apoptotic cells in CD11c$^+$ cells were analyzed by FCM (C and D). The values were the means ± SD (n = 9). *$P < 0.05$, **$P < 0.01$.

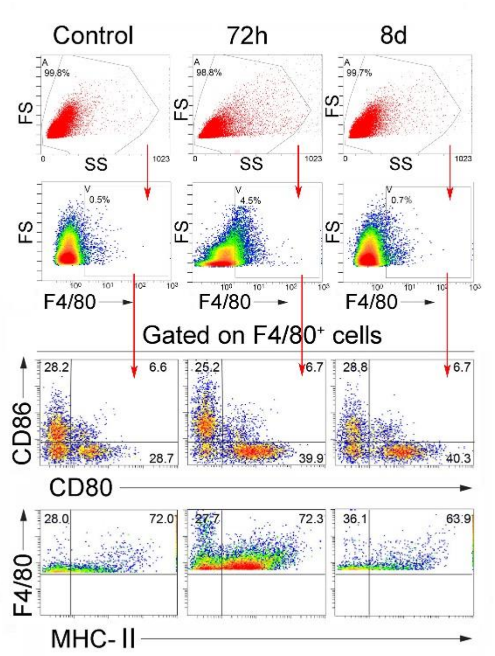

Supplementary Figure 3. FCM analysis strategy diagram of CD86, CD80 and MHC class II expression on F4/80^{+} macrophages.

The C57BL/6 WT mice (female, n=9) were treated with 5 mg/kg LPS *i.p.*. At indicated time points (0 day, 72 hours and 8 days), thymocytes from these mice were collected, and the live status of thymocytes was analyzed by trypan blue staining. Under the condition of the percentage of live cells was above 90%, the percentages of F4/80^{+} macrophages in thymocytes were analyzed by FCM. And then the percentage of CD86, CD80 and MHC class II on F4/80^{+} macrophages were analyzed based the gating on F4/80^{+} macrophages.
